# RANTES and IL-6 cooperate in inducing a more aggressive phenotype in breast cancer cells

**DOI:** 10.18632/oncotarget.24784

**Published:** 2018-04-03

**Authors:** Marianna Gallo, Daniela Frezzetti, Cristin Roma, Nicoletta Chicchinelli, Antonio Barbieri, Claudio Arra, Giosuè Scognamiglio, Gerardo Botti, Antonella De Luca, Nicola Normanno

**Affiliations:** ^1^ Cell Biology and Biotherapy Unit, Istituto Nazionale Tumori-IRCCS-“Fondazione G. Pascale”, Naples, Italy; ^2^ Animal Facility, Istituto Nazionale Tumori-IRCCS-“Fondazione G. Pascale”, Naples, Italy; ^3^ Surgical Pathology Unit, Istituto Nazionale Tumori-IRCCS-“Fondazione G. Pascale”, Naples, Italy

**Keywords:** breast cancer, RANTES/CCL5, IL6, metastasis, tumor microenvironment

## Abstract

Both the CC chemokine ligand 5 (CCL5/RANTES) and interleukin-6 (IL-6), released by mesenchymal stem cells (MSCs) as well as by neoplastic cells, promote breast cancer cell progression through autocrine and paracrine mechanisms. In order to assess the effects of the simultaneous overexpression of RANTES and IL-6 on the tumor cell phenotype, we overexpressed both proteins in MCF-7 and MDA-MB-231 human breast cancer cell lines. MCF-7 cells co-expressing RANTES and IL-6 had a greater ability to form colonies in soft agar, compared to cells overexpressing RANTES or IL-6. In addition, both MCF-7 and MDA-MB-231 clones co-expressing RANTES and IL-6 showed a significantly higher ability to migrate and to invade. The analysis of phosphorylated ERK1/2, AKT and STAT3 signal transduction proteins revealed that several signaling pathways are simultaneously activated in cells overexpressing both factors. Finally, the overexpression of RANTES and IL-6 in MCF-7 cells significantly increased the *in vivo* tumor growth. Collectively, our data suggest that the simultaneous expression of IL-6 and RANTES produces a more aggressive phenotype in breast cancer cells and provide evidence that IL-6 and RANTES might represent potential targets for novel therapeutic strategies aimed to block the tumor-stroma interaction.

## INTRODUCTION

Cancer progression towards metastasis is a multistep process in which tumor cells escape from the primary tumor site and colonize distant organs [1]. During metastatic progression, cancer cells interact with cells of the tumor microenvironment in order to create an environment suitable for adhesion and invasion [2]. A variety of soluble cytokines and chemokines, released by cells of the tumor microenvironment including mesenchymal stem cells (MSCs) as well as by neoplastic cells, acts through autocrine and paracrine mechanisms and promotes the ability of cancer cells to metastasize [1, 3]. Among these factors, we previously demonstrated that MSCs produced significant amounts of the CC chemokine ligand 5 (RANTES/CCL5, hereafter referred to as RANTES) and interleukin (IL)-6 [4].

Evidence suggests that RANTES and IL-6 play a relevant role in the pathogenesis and progression of breast cancer. RANTES is an inflammatory chemokine, frequently expressed in breast cancer cells [5]. The presence of high levels of RANTES has been correlated with a more advanced stage of the disease in breast cancer patients [6, 7]. In addition, a multivariate analysis indicated that RANTES might be considered as a predictor of risk of disease progression in stage II breast cancer [8]. Several different *in vitro* studies demonstrated that RANTES, either secreted by tumor cells or by MSCs, promotes breast cancer progression. In this regard, tumor-derived RANTES was found to contribute to the metastatic potential of murine mammary carcinomas [9]. A pivotal study also showed that MSC-derived RANTES acts in a paracrine fashion on human breast cancer cells to enhance their motility, invasion and ability to form metastasis [10]. Recently, we confirmed that RANTES is able to induce the migration of human breast cancer cell lines representative of different breast carcinoma subtypes [4].

The inflammatory cytokine IL-6 is implicated in the pathogenesis and progression of many human cancers, through the activation of several signal transduction pathways, including JAK/STAT3, RAS/ERK and PI3K/AKT signaling cascades [11]. Elevated levels of serum IL-6 are a biomarker of poor prognosis in most malignancies, including breast cancer [12, 13]. In preclinical studies, IL-6 has been demonstrated to promote breast cancer cell migration in cooperation with EGFR signaling, through an autocrine loop involving EGF family ligands that contribute with IL-6 in inducing ERK activation [14]. In addition, IL-6 was found to significantly induce the *in vitro* and *in vivo* growth of estrogen receptor (ER) positive breast cancer cells [15].The ability of IL-6 to promote breast cancer cell migration was also confirmed by our group [4]. More importantly, we recently reported that recombinant IL-6 cooperates with other factors, such as recombinant VEGFA, in sustaining breast cancer cell migration [16]. In fact, both VEGFA and IL-6 were able to significantly increase the ability to migrate of different breast cancer cell lines, with the combination of the two factors showing a greater effect as compared to treatment with a single protein. Analogously, the combination of anti-VEGFA and anti-IL-6 blocking antibodies was more efficient in inhibiting the spontaneous migration of breast cancer cells as compared with a single antibody.

The above-summarized findings suggest that different secreted factors might cooperate in sustaining the growth and progression of breast cancer cells through autocrine and paracrine circuits. Despite it has been demonstrated that both IL-6 and RANTES favor breast cancer proliferation and migration, the effects of the simultaneous overexpression of RANTES and IL-6 on breast cancer cells’ phenotype have not been explored. For this purpose, we co-expressed both proteins in breast cancer cells and analyzed the ability of stable transfectants to proliferate, migrate, invade and grow *in vivo* in nude mice.

## RESULTS

### Isolation of clones of breast cancer cells with stable co-expression of IL-6 and RANTES

We addressed the role of the simultaneous expression of IL-6 and RANTES in breast cancer progression using two cell lines belonging to different subtypes of breast cancer, the luminal cell line MCF-7, which has a low metastatic potential and invasive ability, and MDA-MB-231 cells, a basal breast cancer cell line with a high metastatic potential.

MCF-7 cells express higher basal levels of RANTES than MDA-MB-231 cells [6, 17], whereas MDA-MB-231 cells produce elevated levels of IL-6 as compared with MCF-7 cells that show undetectable levels of IL-6 [15].

In agreement with these findings, we detected in the conditioned media from MCF-7 cells very low levels of IL-6 and moderate levels of RANTES, whereas the conditioned media from MDA-MB-231 contained high levels of IL-6 and low levels of RANTES (Supplementary Table 1).

MCF-7 and MDA-MB-231 cells were stably co-transfected with two expression vectors containing the human IL-6 and RANTES coding sequences or with the corresponding empty vectors. After 2 weeks of selection with G-418 and zeocin, MCF-7^RANTES+IL6^ and MDA-MB-231^RANTES+IL6^ cells stably overexpressing both IL-6 and RANTES, and the corresponding cell lines carrying the empty vectors were obtained. MCF-7 and MDA-MB-231 clones containing IL-6 or RANTES alone were also generated. To confirm the expression of IL-6 and/or RANTES in stable transfectants, we measured the levels of the secreted protein in conditioned media from all clones using the XMAP Bio-Plex Cytokine arrays. The analysis revealed that MCF-7^RANTES+IL6^ and MDA-MB-231^RANTES+IL6^ cells secreted higher amounts of IL-6 and RANTES than cells transfected with empty vectors (Table 1). Higher levels of secreted IL-6 were also observed in MCF-7^IL6^ and MDA-MB-231^IL6^ cells, as compared with cells transfected with the corresponding empty vector. Similarly, MCF-7^RANTES^ and MDA-MB-231^RANTES^ cells expressed higher levels of RANTES, compared to control cells (Table 1). The differences observed in terms of amount of secreted proteins between parental cells and cells transfected with empty vectors were due to clonal selection and did not affect the biological properties (i.e. proliferation and migration ability) of control cells, transfected with one or two empty vectors compared with parental cells (Supplementary Figure 1A and data not shown).

**Table 1:** Levels of expression of RANTES and IL-6 in conditioned media from stable transfectants

Cell line	RANTES (pg/48 h/10^5^ cells) (mean ± SEM)	*P* value	IL-6 (pg/48 h/10^5^ cells) (mean ± SEM)	*P* value
MCF-7^EVz^	3.44 ± 0.06		1.06 ± 0.04	
MCF-7^IL6^	11.09 ± 0.12	*0.0003*	1574.36 ± 26.97	*0.0003*
MCF-7^EVn^	2.47 ± 0.29		0.80 ± 0.025	
MCF-7^RANTES^	55.42 ± 1.41	*0.0007*	0.17 ± 0.039	*0.0055*
MCF-7^EVn+z^	4.53 ± 0.03		0.60 ± 0.20	
MCF-7^RANTES+IL6^	19.75 ± 0.43	*0.0008*	517.37 ± 8.80	*0.0003*
MDA-MB-231^EVz^	0.41 ± 0.007		1273.01 ± 40.69	
MDA-MB-231^IL6^	0.52 ± 0.002	*0.0111*	1690.70 ± 2.49	*0.0094*
MDA-MB-231^EVn^	0.52 ± 0.005		1003.23 ± 21.54	
MDA-MB-231^RANTES^	161.76 ± 12.93	*0.0063*	849.57 ± 22.70	*0.0391*
MDA-MB-231^EVn+z^	0.55 ± 0.31		808.35 ± 19.76	
MDA-MB-231^RANTES+IL6^	163.35 ± 5.02	*0.0009*	1908.05 ± 34.54	*0.0013*

EV = Empty vector; *n* = neomycin; z = zeocin.

values were referred to 1 × 10^5^ cells as determined on the harvesting day, 48 h after seeding. The data presented above were obtained from three independent measurements. The *P* values were calculated with the Student’s *t*-test (cells overexpressing RANTES or/and IL-6 versus cells transfected with their respective empty vectors).

**Figure 1:**
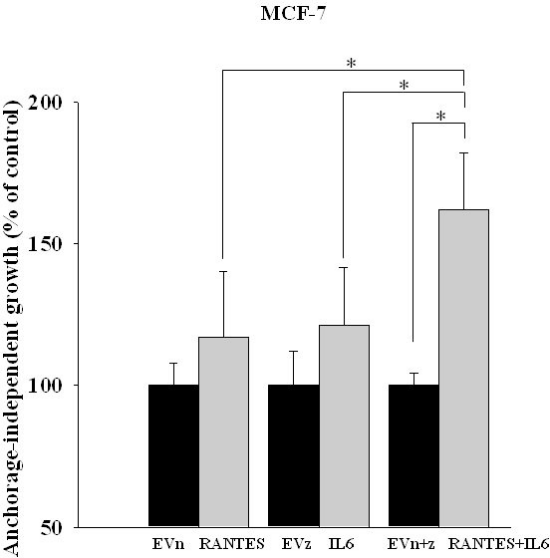
Anchorage-independent growth of clones of MCF-7 cells transfected with RANTES and/or IL-6 The growth in soft agar of MCF-7^RANTES^, MCF-7^IL6^, MCF-7^RANTES+IL6^ and control cells was performed as described in the Material and Methods (^*^P < 0.05; Student’s *t*-test). EVn: cells transfected with the empty vector (neomycin); EVz: cells transfected with the empty vector (zeocin); EVn+z: cells transfected with both empty vectors.

To assess the effects of the simultaneous expression of IL-6 and RANTES on breast cancer cell proliferation, we analyzed the anchorage-dependent and -independent growth of stable transfectants. The overexpression of IL-6 and/or RANTES did not promote the anchorage-dependent growth of MCF-7 and MDA-MB-231 cells compared to their respective control cells (Supplementary Figure 1B). In these experiments, MCF-7 and MDA-MB-231 cells transfected with empty vectors showed a proliferation index similar to parental cells (Supplementary Figure 1A). In contrast, we observed that the ability to form colonies in soft agar was significantly increased in MCF-7^RANTES+IL6^ cell line, as compared with clones overexpressing IL-6 or RANTES alone (Figure 1). The single or combined expression of IL-6 and RANTES did not affect the anchorage-independent growth of MDA-MB-231 cells in soft agar colony formation assays (data not shown).

### RANTES and IL-6 promote breast cancer cell migration and invasion

**Figure 2:**
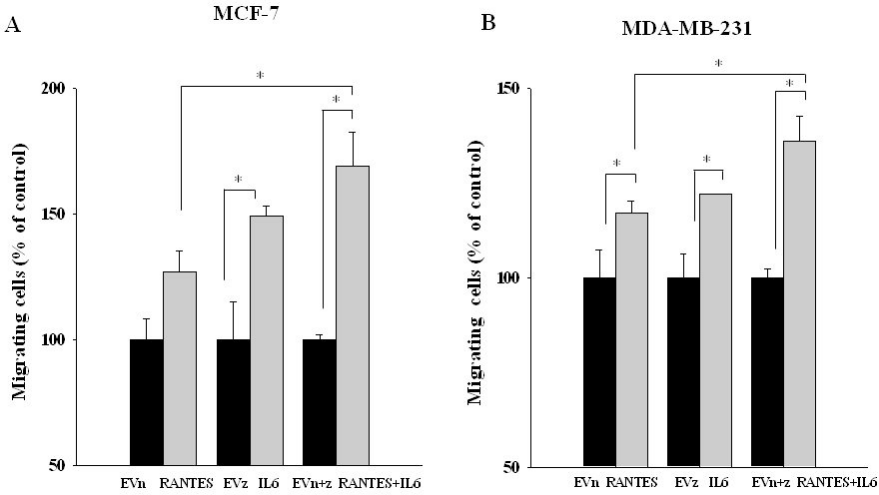
Migratory ability of MCF-7 and MDA-MB-231 clones transfected with RANTES and/or IL-6 MCF-7 (**A**) and MDA-MB-231 (**B**) stable clones were seeded in the inserts and allowed to migrate for 20 h through a fibronectin-coated membrane toward serum containing medium (^*^P < 0.05; Student’s *t*-test). EVn: cells transfected with the empty vector (neomycin); EVz: cells transfected with the empty vector (zeocin); EVn+z: cells transfected with both empty vectors.

**Figure 3:**
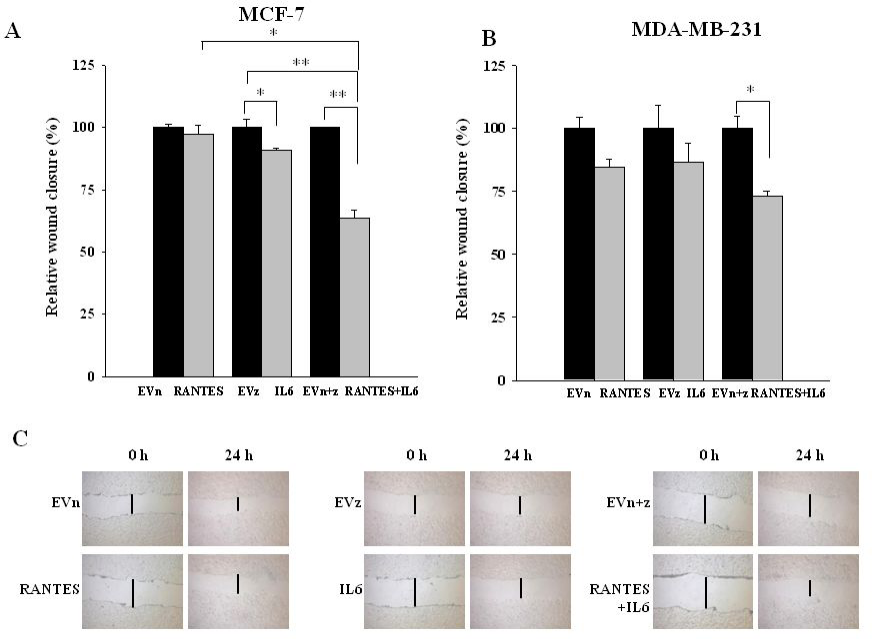
Effects of the overexpression of RANTES and/or IL-6 on wound healing in MCF-7 and MDA-MB-231 cells Wound healing assays were performed on (**A**) MCF-7^RANTES^, MCF-7^IL6^, MCF-7^RANTES+IL6^ cell lines and control cells and (**B**) MDA-MB-231^RANTES^, MDA-MB-231^IL6^, MDA-MB-231^RANTES+IL6^ cell lines and their respective control cells (^*^P < 0.05; ^**^P < 0.005; Student’s *t*-test). (**C**) Representative images of the wound tracks at time point zero and after 24 h were shown for MCF-7 derived clones (magnification of 20X). EVn: cells transfected with the empty vector (neomycin); EVz: cells transfected with the empty vector (zeocin); EVn+z: cells transfected with both empty vectors.

In order to evaluate whether the combined expression of RANTES and IL-6 is able to induce a more aggressive tumor phenotype, we analyzed the migratory and invasive ability of MCF-7^RANTES+IL6^ and MDA-MB-231^RANTES+IL6^ cell lines. Previous studies demonstrated that both recombinant RANTES or IL-6 as single factors promote cell migration [16, 18]. When RANTES and IL-6 were simultaneously expressed, a more significant increase in the migration of both MCF-7 and MDA-MB-231 cells was observed, as compared to breast cancer cells transfected with RANTES or with IL-6 alone (Figure 2A and 2B). Accordingly with these data, the co-expression of RANTES and IL-6 induced a faster wound closure compared to the single factors in wound healing assays in both MCF-7 and MDA-MB-231 cells (Figures 3A–3C). No differences in the wound closure were observed between breast cancer cells transfected with empty vectors and parental cells (data not shown).

**Figure 4:**
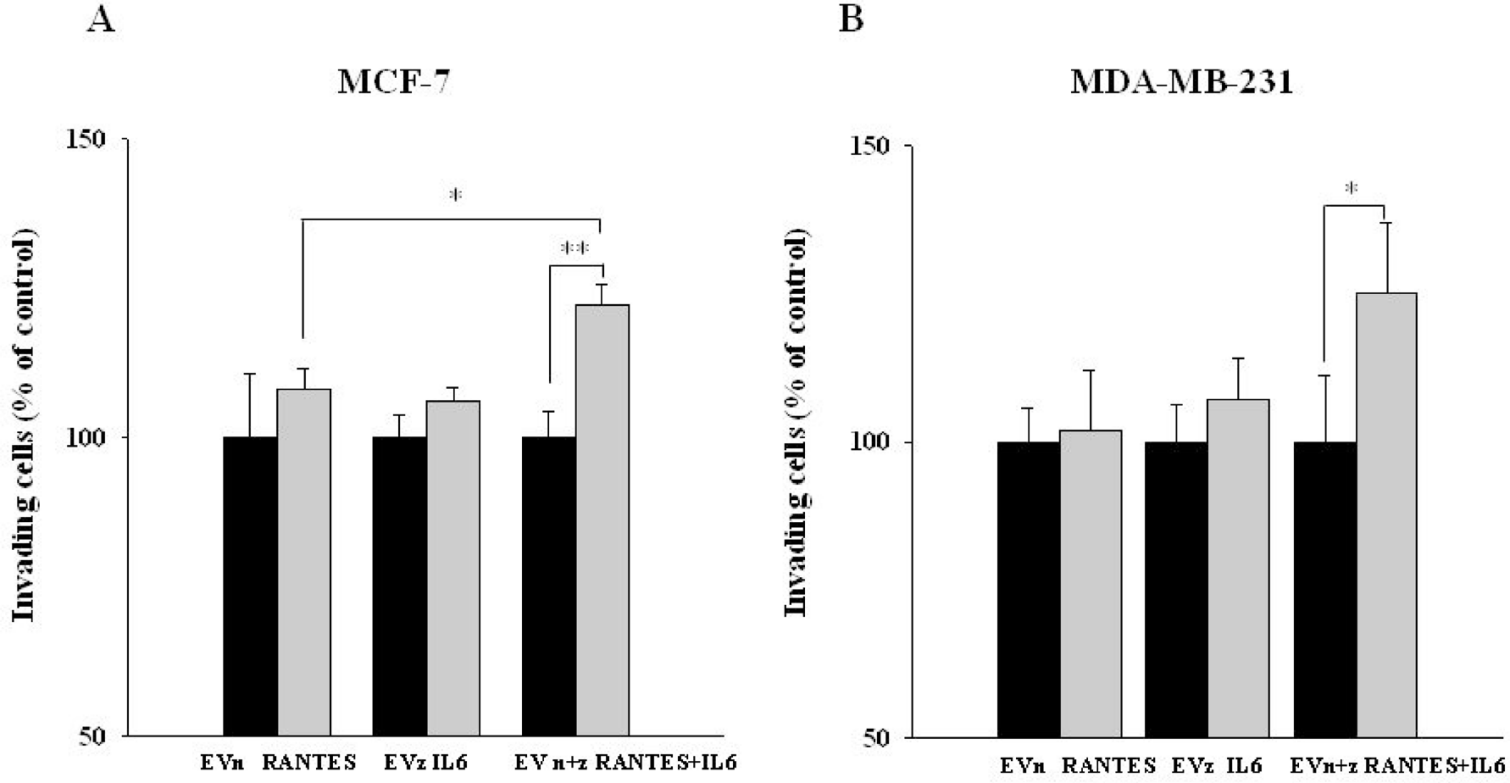
Invasive ability of MCF-7 and MDA-MB-231-derived stable clones overexpressing RANTES and/or IL-6 The invasive ability of (**A**) MCF-7^RANTES^, MCF-7^IL6^, MCF-7^RANTES+IL6^ cell lines and control cells and (**B**) MDA-MB-231^RANTES^, MDA-MB-231^IL6^, MDA-MB-231^RANTES+IL6^ cell lines and their respective control cells, was determined using a Boyden chamber-based colorimetric assay (^*^P < 0.05; ^**^P < 0.005; Student’s *t*-test). EVn: cells transfected with the empty vector (neomycin); EVz: cells transfected with the empty vector (zeocin); EVn+z: cells transfected with both empty vectors.

We next assessed the effects of IL-6 and/or RANTES overexpression on the ability of MCF-7 and MDA-MB-231 cells to invade through a Matrigel membrane. Again, the co-expression of the two factors induced a more significant increase of the invasive ability of breast cancer cells as compared with the expression of a single factor (Figure 4A and 4B).

### Effects of the simultaneous overexpression of RANTES and IL-6 on the activation of signaling pathways

**Figure 5:**
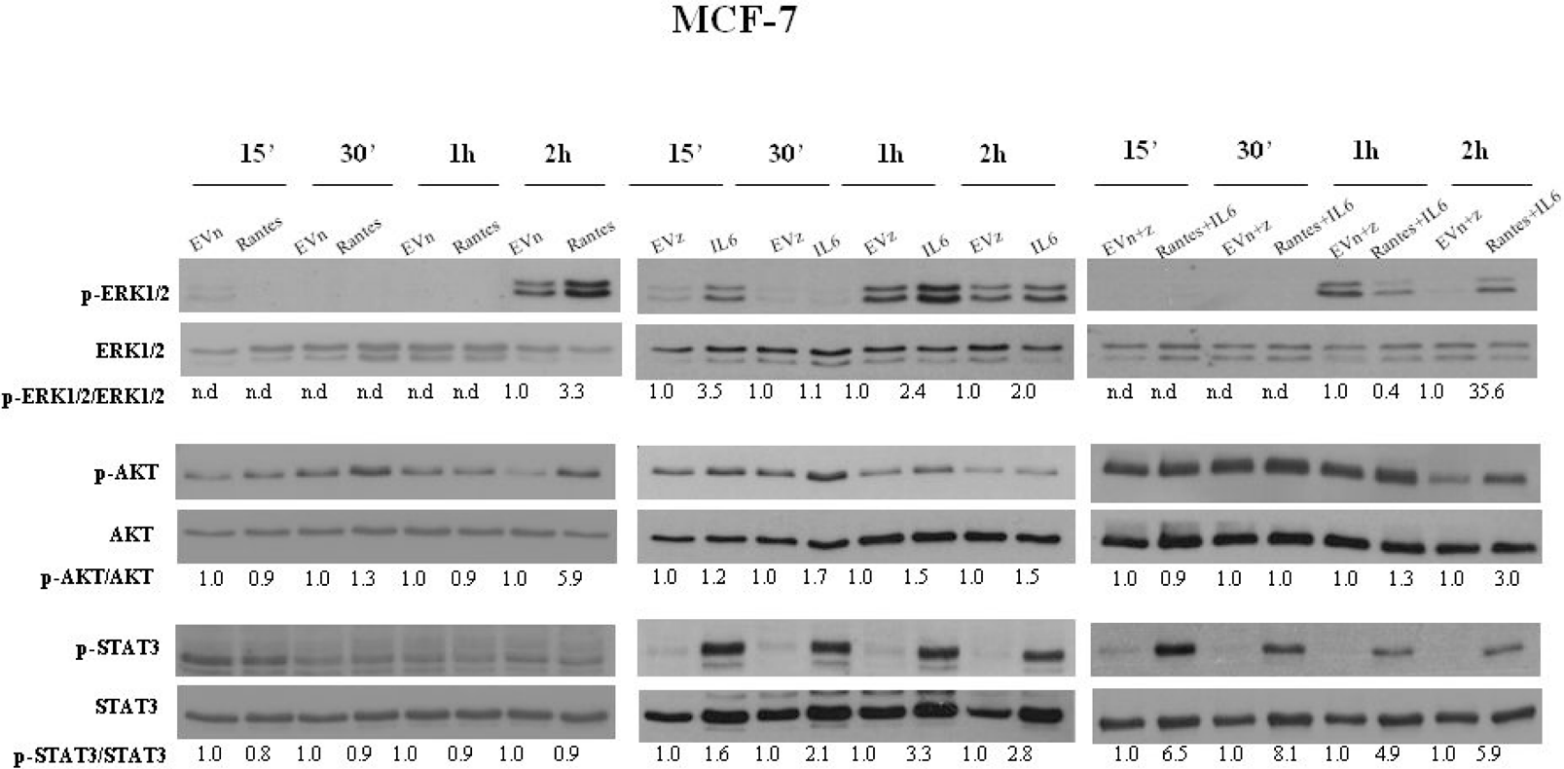
Analysis of the activation of ERK1/2, AKT and STAT3 in MCF-7 stable clones Western Blot analysis for the expression of phosphorylated and total ERK1/2, AKT and STAT3 in MCF-7 cells overexpressing RANTES and/or IL-6 at the indicated time points. Densitometric value ratios for pERK1/2/total ERK1/2, pAKT/total AKT and pSTAT3/total STAT3 were shown for each panel. EVn: cells transfected with the empty vector (neomycin); EVz: cells transfected with the empty vector (zeocin); EVn+z: cells transfected with both empty vectors.

**Figure 6:**
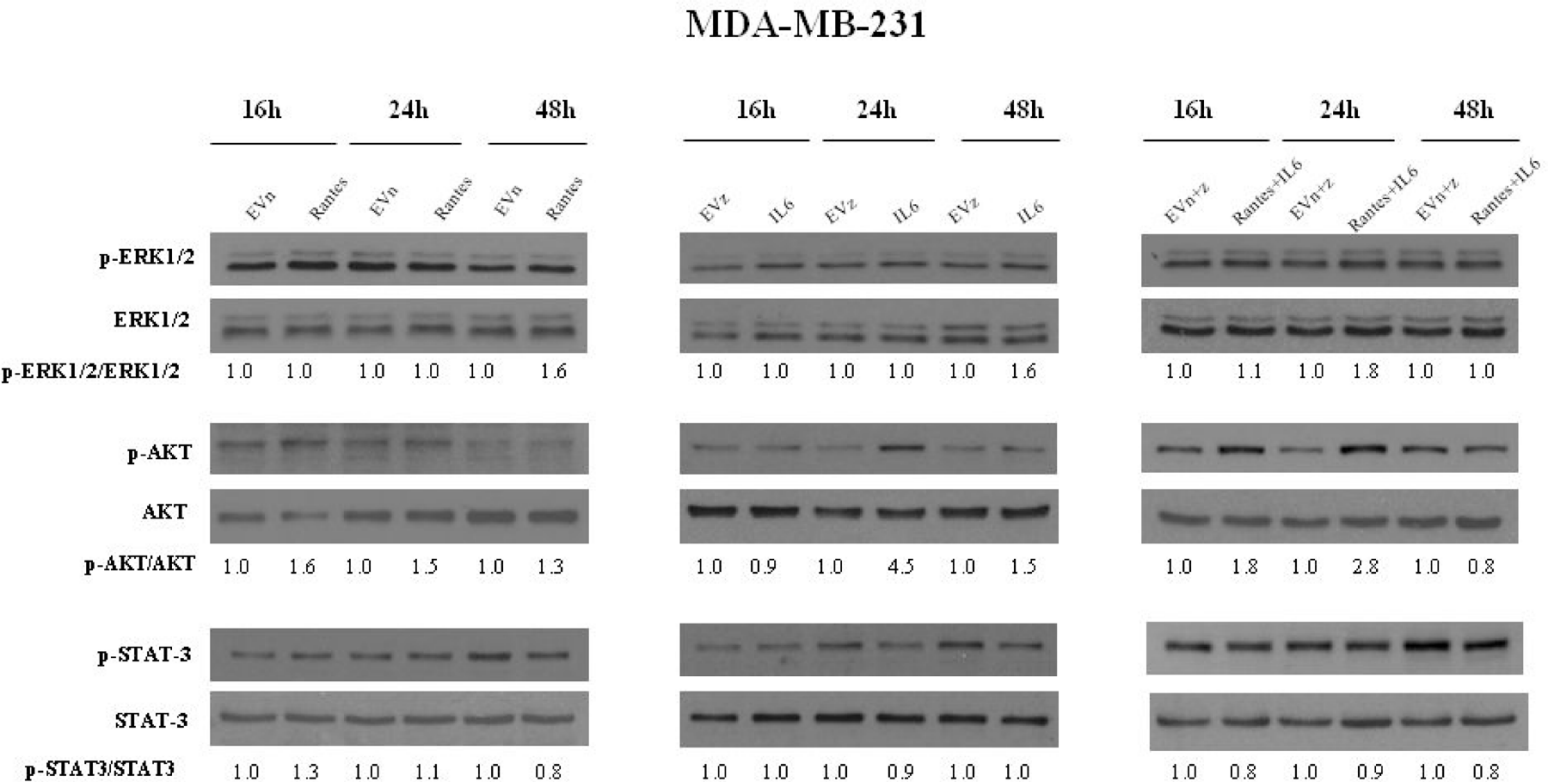
Analysis of the activation of ERK1/2, AKT and STAT3 in MDA-MB-231 stable clones Western Blot analysis for the expression of phosphorylated and total ERK1/2, AKT and STAT3 in MDA-MB-231 cells overexpressing RANTES and/or IL-6 at the indicated time points. Densitometric value ratios for pERK1/2/total ERK1/2, pAKT/total AKT and pSTAT3/total STAT3 were shown for each panel. EVn: cells transfected with the empty vector (neomycin); EVz: cells transfected with the empty vector (zeocin); EVn+z: cells transfected with both empty vectors.

To address the molecular mechanisms through which IL-6 and RANTES promote breast cancer cell migration and invasion, we analyzed their effects on the activation of ERK1/2, AKT and STAT3 signal transduction proteins at different time points (Figures 5 and 6). In MCF-7 transfected clones, we observed that RANTES and IL-6 modulated signaling proteins within 2 h from the passage in serum free medium. Since in MDA-MB-231-derived clones no effects of signaling transduction proteins were observed within this time (Supplementary Figure 2), we analyzed the effects of RANTES and IL-6 at longer time points.

We found that the overexpression of RANTES alone produced a marked activation of ERK1/2 and AKT at 2 h in MCF-7 cells (Figure 5) and a slight activation of AKT at 16 h and of ERK1/2 at 48 h in MDA-MB-231 cells (Figure 6). IL-6 induced in MCF-7 cells a strong and persistent activation of STAT3 and ERK1/2 and a slight activation of AKT at 30’ (Figure 5). In MDA-MB-231^IL6^ cells, a peak of AKT phosphorylation at 24 h and a slight activation of ERK1/2 at 48 h were observed (Figure 6). The simultaneous expression of RANTES and IL-6 produced in MCF-7 cells a marked increase of the phosphorylation of ERK1/2 and AKT at 2 hours and a persistent activation of STAT3, whereas in MDA-MB-231^RANTES+IL6^ cells a marked phosphorylation of ERK1/2 at 24 h and of AKT at 16 and 24 h were observed (Figures 5 and 6). The activation of Focal Adhesion Kinase (FAK) and Paxillin signaling proteins involved in cell adhesion and migration has been also investigated. We did not find differences between single and double recombinants in both cell lines (data not shown).

These data suggest that several signaling pathways involved in cancer progression might be simultaneously activated when RANTES and IL-6 are co-expressed in breast cancer cells.

### IL-6 and RANTES promote MCF-7 *in vivo* growth

**Figure 7:**
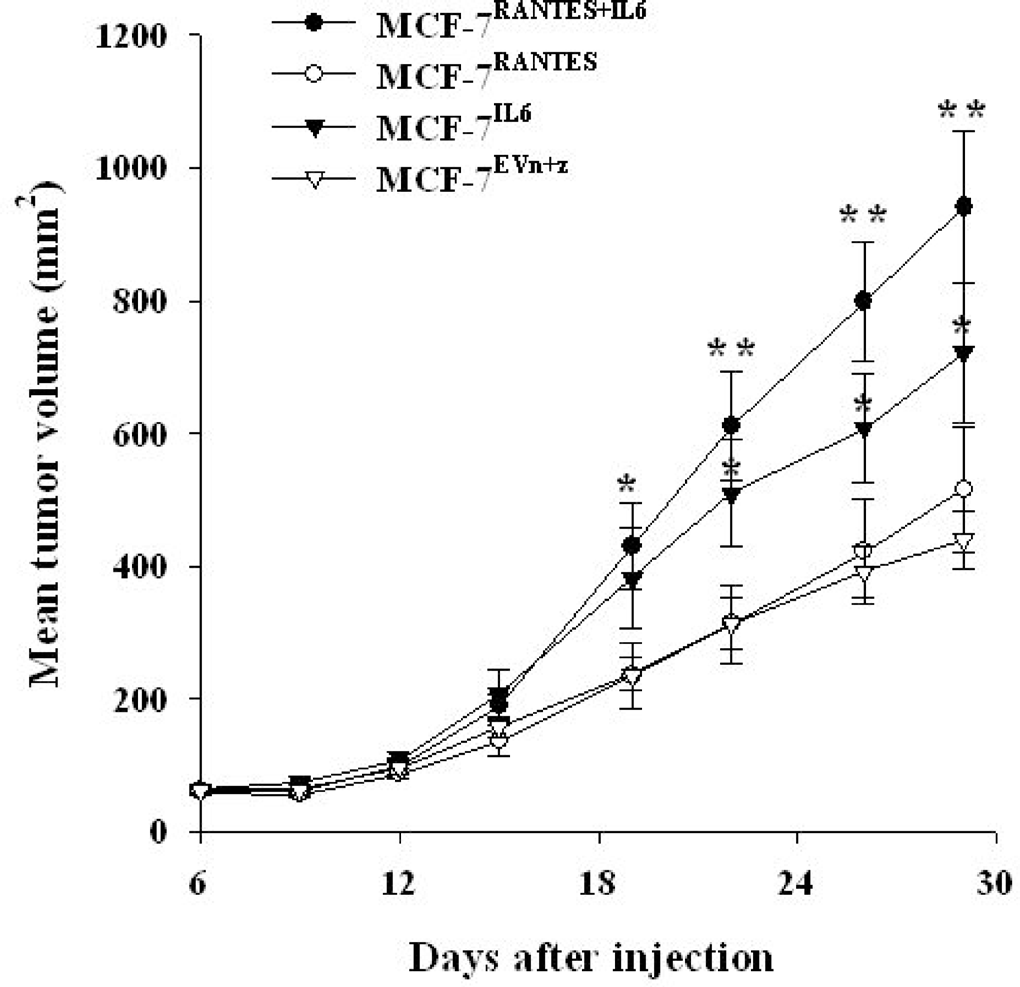
IL-6 and RANTES promote MCF-7 tumor growth *in vivo* MCF-7 stable transfectants were orthotopically injected in nude mice under estrogen supplementation. Six mice were injected with MCF-7^RANTES^, MCF-7^IL6^ or MCF-7^RANTES+IL6^ cells, and 8 mice were injected with MCF-7^EVn+z^ cells. Data are expressed as the mean tumor volume values ± SE (^*^P < 0.05; ^**^P < 0.005 Student’s *t*-test for comparison between MCF-7 stable transfectants versus MCF-7 transfected with both empty vectors at the different time points). EV: empty vector; n: neomycin; z: zeocin.

Finally, we analyzed the effect of RANTES and IL6 overexpression on tumor growth of breast cancer cells *in vivo*. For this purpose, we injected MCF-7 and MDA-MB-231 cells transfected with IL6 or/and RANTES and control cells transfected with both empty vectors into the mammary fat pad of nude mice. Consistently with other reports, we observed that the overexpression of RANTES did not affect the tumorigenesis of MCF-7 cells and MDA-MB-231 cells, whereas in MCF-7 cells IL-6 increased the rate of tumor growth as compared with control cells transfected with empty vectors [10, 15]. However, the simultaneous overexpression of RANTES and IL-6 in MCF-7 cells produced a more significant increase in tumor growth *in vivo* confirming that these factors cooperate to induce a more aggressive phenotype (Figure 7). Differently, in MDA-MB-231 cells the expression of both RANTES and IL-6 cells did not produce a significant increase of the tumor growth *in vivo* (Supplementary Figure 3).These results are in agreement with the less pronounced *in vitro* effects of cytokine overexpression in MDA-MB-231 cells as compared with MCF-7 cells. As control, we verified that MCF-7 and MDA-MB-231 clones retained their ability to express the transfected cytokines *in vivo*, by immunohistochemical analysis of RANTES expression in mammary tumors (Supplementary Figure 4).

No visceral metastases were observed in mice injected with control or cytokine/s overexpressing MCF-7 cells. Enlarged inguinal lymph nodes in proximity of the mammary tumors were observed in 2/8 (25%) of control mice, and in 3/6 (50%), 1/6 (16%) and 5/6 (83%) of mice injected with MCF-7^IL6^, MCF-7^RANTES^ or MCF-7^IL6+RANTES^ cells, respectively. However, the presence of metastases was confirmed in one lymph node from MCF-7^RANTES^ injected mice and in two lymph nodes from two different mice carrying xenografts of MCF-7^IL6+RANTES^ cells. No lymph nodal metastases were detected in mice injected with MCF-7^IL6^ or MCF7^EVn+z^ cells (Supplementary Figure 5). Finally, in mice injected with MDA-MB-231 stable transfectants, no differences in terms of number of metastases have been observed (data not shown).

## DISCUSSION

The present study confirmed the potential role in the progression of breast cancer of RANTES and IL-6. Indeed, we found that both factors were able to promote the migration of breast cancer cells. In this regard, we and other groups have previously demonstrated that IL-6 is able to promote the migration and the invasion of both ER-positive and HER2-positive breast cancer cells [14, 16, 19]. However, in agreement with our previous findings, we did not observe significant effects of IL-6 on anchorage-dependent and -independent breast cancer cell growth [16]. In this respect, studies in which IL-6 was shown to promote breast cancer cell proliferation used particular conditions in growth assays, such as 3D assays in which cells were embedded in a basement membrane extract, probably enriched with different growth factors [15, 20]. Similar results have been described for RANTES that did not increase the proliferation of MDA-MB-231 cells both in anchorage-dependent and independent assays, although it was able to significantly increase migration and invasion [10]. The key role of RANTES in the pathogenesis of triple negative breast cancer was confirmed by the observation that β-catenin-induced RANTES was essential for the formation of spheroid colonies by MDA-MB-231 cells [21]. Interestingly, in MCF-7 cells, recombinant RANTES was found to induce proliferation only when cells were transfected with its receptor CCR5 [22].

Although the role of these factors has been well established, our data demonstrate for the first time that RANTES and IL-6 cooperate in promoting the aggressiveness of breast cancer cells. In fact, the effects of the combination of the two factors on migration, invasion and *in vivo* growth were more significant as compared with a single growth factor. In this respect, we have previously demonstrated that VEGFA and IL-6 cooperate in inducing the migration of breast cancer cells. We have also shown that a number of cytokines and chemokines are likely to be upregulated within the tumor microenvironment due to complex interactions between tumor and stromal cells that involve different signaling pathways including the EGFR [23, 24]. Taken together, these findings strongly suggest that a network of functionally related cytokines is involved in determining the aggressiveness of breast cancer cells.

The effects of RANTES and IL-6 co-expression on the phenotype of breast cancer cells were more marked in MCF-7 cells, a poorly aggressive ER positive luminal cell line, as compared with the highly aggressive basal MDA-MB-231 cell line. In particular, the co-expression of the two factors increased the anchorage-independent growth in MCF-7 cells, but not in MDA-MB-231 cells. Nevertheless, several lines of evidence suggest that RANTES and IL-6 also play a fundamental role in the progression of triple negative breast cancer. Indeed, a recent study demonstrated that inducible IκB kinase-related (IKK-related) kinase IKBKE promotes in triple negative breast cancer cells the expression of IL-6 and CCL5, which in turn sustain the proliferation and migration of breast cancer cells in 3D culture [25]. Therefore, overexpression of both factors occurs in triple negative breast cancer cells within a signaling pathway that links tumor growth to inflammation.

The STAT3, RAS/MEK/ERK and PI3K/AKT signaling pathways are often up-regulated in breast cancer cells and are fundamental to proliferation, differentiation, migration and invasion. It has previously suggested that IL-6 stimulated the proliferation of breast cancer cells mainly through the activation of STAT3 and cell migration through the activation of the RAS/MEK/ERK and PI3K/AKT signaling pathways [14]. We observed that the simultaneous expression of RANTES and IL-6 in MCF-7 cells induced a strong activation of STAT3 that might stimulate the anchorage-independent growth, whereas in MDA-MB-231^RANTES+IL6^ cells, in which STAT3 was constitutively activated, no effects on cell proliferation were observed. The activation of ERK1/2 and AKT in MCF-7^RANTES+IL6^ and MDA-MB-231^RANTES+IL6^ clones might explain the effects observed on migration and invasive ability observed in double transfectant clones in both cell lines.

Our results might have clinical implications. Metastatic disease is the principal cause of death for the majority of breast cancer patients. Currently, few therapies that specifically target metastatic breast cancer are available. Drugs that target tumor-stromal interactions might represent an attractive therapeutic strategy in metastatic breast cancer [26]. In this regard, blockade of RANTES and IL-6 that can in turn interact with cancer cells or cells of the tumor microenvironment might prevent breast cancer progression.

In conclusion, our data suggest that the simultaneous expression of IL-6 and RANTES produces a more aggressive phenotype in breast cancer cells. This observation might be useful for the identification of potential targets for novel therapeutic strategies aimed to prevent breast cancer progression through the blockade of the tumor-stroma interaction.

## MATERIALS AND METHODS

### Cell lines

The human breast cancer cell lines MDA-MB-231 and MCF-7 were purchased from the American Type Culture Collection (ATCC, Manassas, VA, USA). MDA-MB-231 cells were routinely grown in RPMI 1640 medium with GlutaMAX supplemented with 10% fetal bovine serum (FBS) (all from Thermo Fisher Scientific, Milan, Italy) at 37° C in a humidified incubator supplemented with 5% carbon dioxide. MCF-7 cells were cultured in a 1:1 mixture of Dulbecco’s modified Eagle’s medium (DMEM)/Ham’s F-12 medium with GlutaMAX supplemented with 10% FBS (Thermo Fisher Scientific).

### RANTES and IL6 expression vectors and stable transfection

The cDNA encoding the full open reading frame (ORF) of human RANTES was cloned into the multiple cloning site between the XhoI and ApaI restriction sites of the expression vector pcDNA3.1 containing the neomycin-resistance gene (Thermo Fisher Scientific) to generate the pcDNA3.1-RANTES expression vector. Similarly, the cDNA encoding the ORF of the human IL-6 was cloned into the expression vector pZeoSV2 containing the zeocin-resistance gene (Thermo Fisher Scientific) between the BamHI and EcoRV restriction sites, to generate the pZeoSV2-IL-6 expression vector. The established RANTES and IL6 expression vectors were subjected to DNA sequencing analysis to confirm the correct insertion of the restriction fragments.

Then, pcDNA3.1-RANTES, pZeoSV2-IL-6 and the corresponding pcDNA3.1 and pZeoSV2 empty vectors were transfected into breast cancer cells using the X-tremeGENE 9 DNA Transfection Reagent (Roche, Milan, Italy) according to the manufacturer’s instructions and after 24 hours, transfected cells were selected with the neomycin analog G-418 and/or zeocin. After 2 weeks of selection, stable clones overexpressing RANTES and/or IL-6 were obtained from MCF-7 and MDA-MB-231 parental cells and designated as follows: MCF-7^RANTES^, MCF-7^IL6^, MCF-7^RANTES+IL6^, MDA-MB-231^RANTES^, MDA-MB-231^IL6^ and MDA-MB-231^RANTES+IL6^. Similarly, clones transfected with the empty vectors were obtained.

### Preparation of conditioned media and immunoassays

Stable transfectants were cultured in serum-free medium for 48 h. Then, the conditioned media were collected, sterile filtered and stored in aliquots at –80° C. The levels of secreted RANTES and IL-6 proteins were assessed using the XMAP Bio-Plex Human Cytokine Multiplex Assay (Bio-Rad, Milan, Italy).

### Anchorage-independent growth assays

MCF-7 (8 × 10^3^ cells/well) and MDA-MB-231 (20 × 10^3^ cells/well) stable transfectants were seeded in 0.3% soft agar (Difco, Detroit, MI, USA) supplemented with complete culture medium. This suspension was layered over 0.8% agar-medium base layer in 24 well culture plates. After 21 days, the colonies were stained with nitro-blue tetrazolium and acquired with a micro-Scopeman camera system (Moritex Europe Ltd.). The number of colonies was determined using the Image-ProPlus software (Media Cybernetics, Rockville, MD, USA).

### Migration assays

Breast cancer cells overexpressing RANTES and/or IL-6 (50 × 10^3^ cells/insert) were seeded in serum-free medium in the inserts and allowed to migrate for 20 h through a fibronectin-coated Boyden chamber using the QCM-FN Haptotaxis Cell Migration Assay-Fibronectin, Colorimetric (Chemicon/Millipore, Milan, Italy) according to manufacturer’s instructions. Serum containing medium was used as chemoattractant in the lower Boyden chambers. Cells that had migrated across the membrane were stained with crystal violet stain solution. The crystal violet stain solution was eluted and the absorbance was read at 540 nm in each well.

### Wound healing assays

Parental and stably transfected breast cancer cells were plated at confluence in 6-well plates (1.2 × 10^6^ cells/well for MCF-7 cells and 1.6 × 10^6^ cells/well for MDA-MB-231 cells). Wounds were performed using sterile pipette tips and cells were washed with PBS. For MCF-7 cell line, cells were then incubated in complete medium for 24 h and the wounds were photographed at time 0 and 24 h after wounding. For MDA-MB-231 cell line, cells were incubated in medium containing 2% FBS and the wounds were photographed at time 0 and 5 h after wounding. Healing was quantified measuring the distance between the edges with the ImageJ software (http://rsb.info.nih.gov/ij/). Three different parts of each wound were analyzed and mean values of distances between the edges were calculated.

### Invasion assays

The invasive ability of stable transfectants was evaluated using the Boyden chamber-based colorimetric assay Cell Invasion Assay Kit (Millipore, Milan, Italy) as previously described [27]. Briefly, cells (50 × 10^3^ cells/insert) were seeded in serum-free medium in the upper chambers and allowed to invade for 20 hours through a matrigel-coated membrane. Medium supplemented with FBS was added as chemoattractant in the lower Boyden chambers. After 20 hours non-invading cells were removed and the invading cells were stained with crystal violet stain solution. The solution was eluted with 10% acetic acid extraction buffer, transferred to wells of a 96-multiwell plate, and the absorbance was read at 595 nm in each well.

### Western Blot analysis

Transfected cells were plated in 60-mm culture dishes in serum containing medium. Then, cells were washed twice with PBS and cultured in serum-free medium for the indicated times.

Western blotting was done using standard procedures. The following antibodies were used: anti-phospho p42/p44 MAPK (ERK1/2) (Thr202/Tyr204); anti-p42/p44 MAPK (ERK1/2); anti-phospho AKT (Ser473); anti-AKT; anti-phospho STAT3 (Tyr705, clone 3E2); anti-STAT3 (Cell Signaling Technology, Danvers, MA,USA); anti-α-tubulin clone DM1A (Sigma-Aldrich, Milan, Italy). Densitometric analysis of the blots was performed using the ImageJ software.

### *In vivo* studies

Six weeks old athymic nude (nu/nu) female mice were purchased from Harlan Laboratories (San Pietro al Natisone, Italy). For xenograft experiments, MCF-7 and MDA-MD-231 cells (7 × 10^6^ cells/mouse and 2 × 10^6^ cells/mouse, respectively) were resuspended in 100 µl of PBS and injected into the abdominal mammary fat pad. For mice injected with MCF-7 cells, two days before the injection, 17β-estradiol pellets were implanted subcutaneously after anaesthesia. Tumor volumes were measured with a caliper twice a week and calculated as follows: V = L × l^2^/2, where L represents the larger and l the smaller tumor diameter, respectively. Mice were sacrificed by cervical dislocation. All the experiments involving animals were approved by the Istituto Pascale Ethic Committee.

### Statistical analysis

Statistical significance was determined using two-tailed Student’s *t*-test and *P* values < 0.05 were considered significant.

## SUPPLEMENTARY MATERIALS FIGURES AND TABLE


