# Supplementary material for: RANTES and IL-6 cooperate in inducing a more aggressive phenotype in breast cancer cells
Source: Oncotarget. 2018 Apr 3;9(25):17543–53. doi: 10.18632/oncotarget.24784 (PMC5915136; doi:10.18632/oncotarget.24784)
Supplement: Supplementary file 1 [file oncotarget-09-17543-s001.pdf]

RANTES and IL-6 cooperate in inducing a more aggressive phenotype in breast cancer cells

SUPPLEMENTARY MATERIALS

Anchorage-dependent growth assay

Parental and stably transfected MCF-7 and MDA-MB-231 cells were seeded in 12-wells cell culture plates (18000 cells/well and 16000 cells/well respectively) in serum containing medium. At the indicated time points, cells were trypsinized and counted with an automated Z1 Coulter Counter (Beckman Coulter, Milan, Italy). Cell proliferation was evaluated counting cells at each time point and calculating the ratio compared to time 0 (proliferation index).

Immunohistochemical analysis of xenografts

Mammary tumors were surgically removed from mice and fixed in 4% formalin, dehydrated through ethanol series, cleared in xylene and embedded in paraffin. For immunohistochemical analysis, 4 µm sections were dewaxed by standard techniques. Antigen retrieval was performed by heat treatment at 90° C for 10 minutes in citrate buffer (pH 8). Endogenous peroxidase activity was quenched using DAKO Peroxidase Blocking Reagent according to manufacturer's protocol (DAKO/Agilent Technology, Milan; Italy) and protein blocking was performed in PBS containing 5% BSA. The anti-RANTES rabbit polyclonal antibody (Abcam, Milan, Italy) was incubated overnight at 4° C at a concentration of 3.3 µg/ml and the EnVision FLEX Visualization Reagent (DAKO) was used as secondary antibody. The chromogenic reaction was carried out using 3,3-diaminobenzidine (DAB) (Abcam) according to manufacturer's instruction. Hematoxylin was used as nuclear counterstain.

Analysis of lymph nodal metastases

Enlarged inguinal lymph nodes localized in the proximity of mammary tumors derived from MCF-7 stably transfected cells were surgically removed and paraffin embedded. For histological analysis, lymph nodes were dissected with a microtome and 3 µm sections were stained with hematoxylin and eosin using an automatic stainer (DAKO/Agilent Technologies). To evaluate the presence of metastases lymph nodes, sections were analyzed by an experienced pathologist.

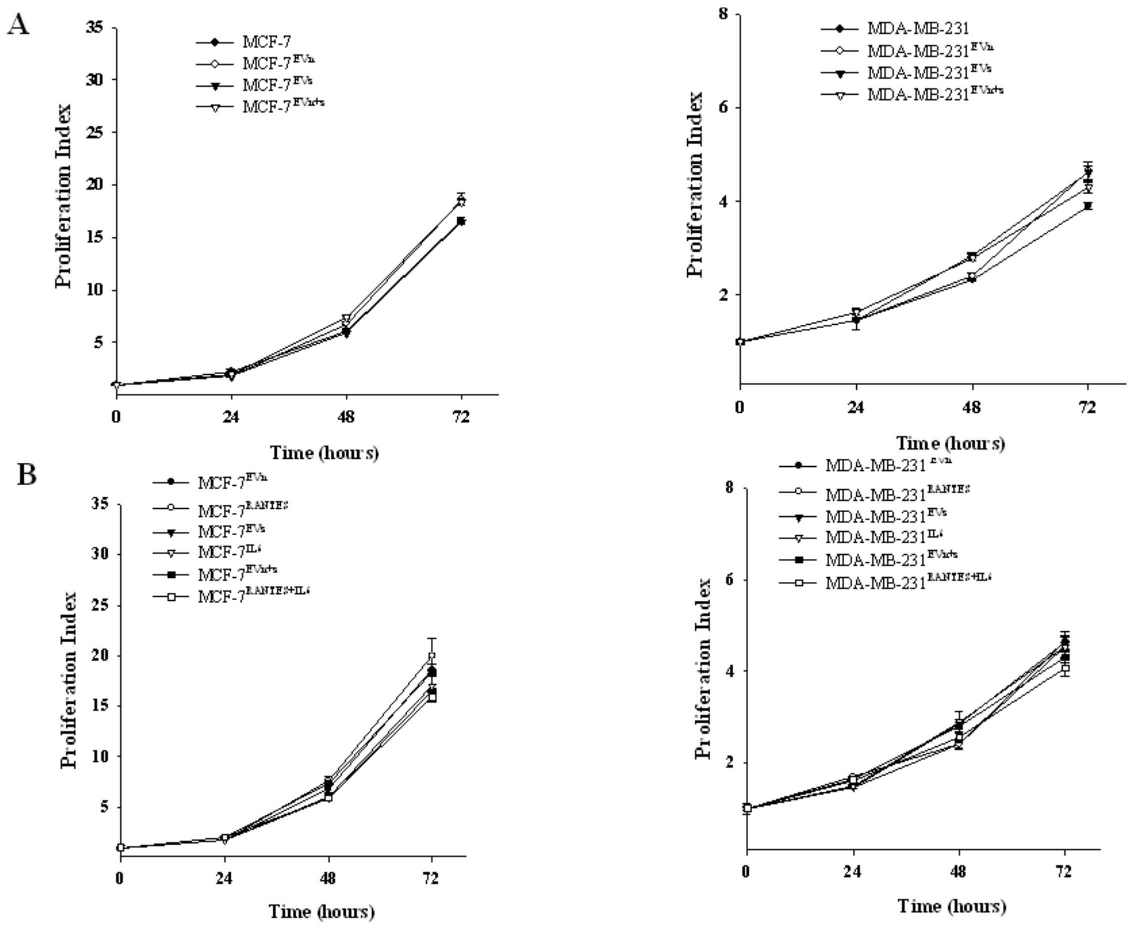

Supplementary Figure 1: Effects of RANTES and/or IL-6 on the anchorage-dependent growth of breast cancer cells. Analysis of proliferation rates was performed (**A**) for parental breast cancer cell lines compared to cells transfected with empty vectors and (**B**) for MCF-7 and MDA-MB-231 cells transfected with RANTES and/or IL-6 and their respective control cells. The proliferation index was calculated at the indicated time points as described in Supplementary Materials and Methods. EVn: cells transfected with the empty vector (neomycin); EVz: cells transfected with the empty vector (zeocin); EVn+z: cells transfected with both empty vectors.

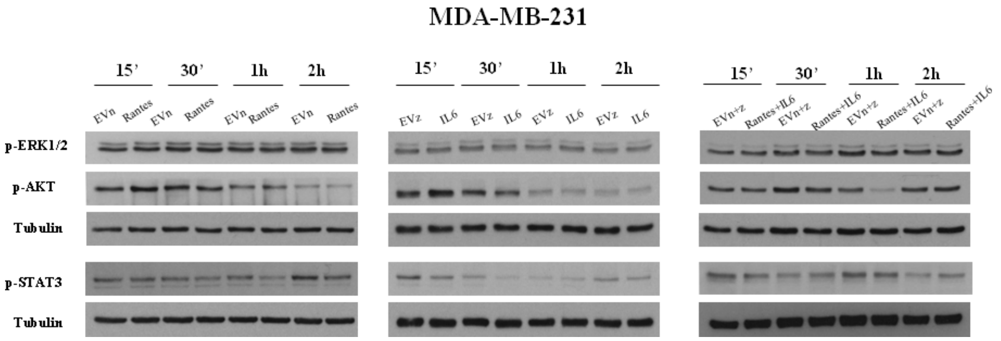

Supplementary Figure 2: Analysis of the activation of ERK1/2, AKT and STAT3 in MDA-MB-231 stable clones. The expression of the activated forms of ERK1/2, AKT and STAT3 in MDA-MB-231 cells overexpressing RANTES and/or IL-6 was analyzed by Western Blot at short times (15 min, 30 min, 1 h and 2 h). The blots were normalized to an α tubulin antibody. EVn: cells transfected with the empty vector (neomycin); EVz: cells transfected with the empty vector (zeocin); EVn+z: cells transfected with both empty vectors.

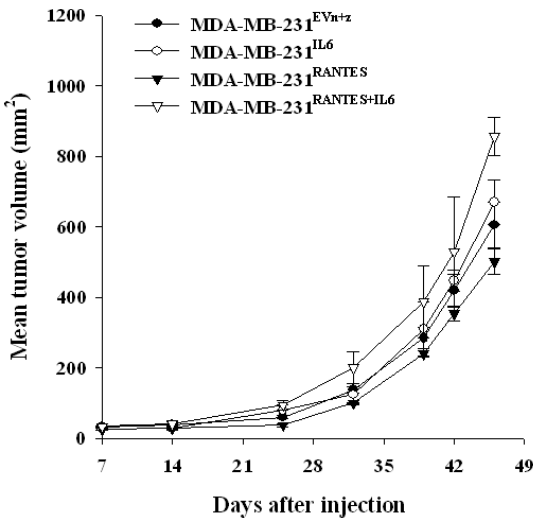

Supplementary Figure 3: Analysis of the effects of RANTES and IL-6 on MDA-MB-231 tumor growth *in vivo*. MDA-MB-231 stable transfectants were orthotopically injected in nude mice (6–9 mice/ cell line). Data are expressed as the mean tumor volume values ± SE. EV: empty vector; n: neomycin; z: zeocin.

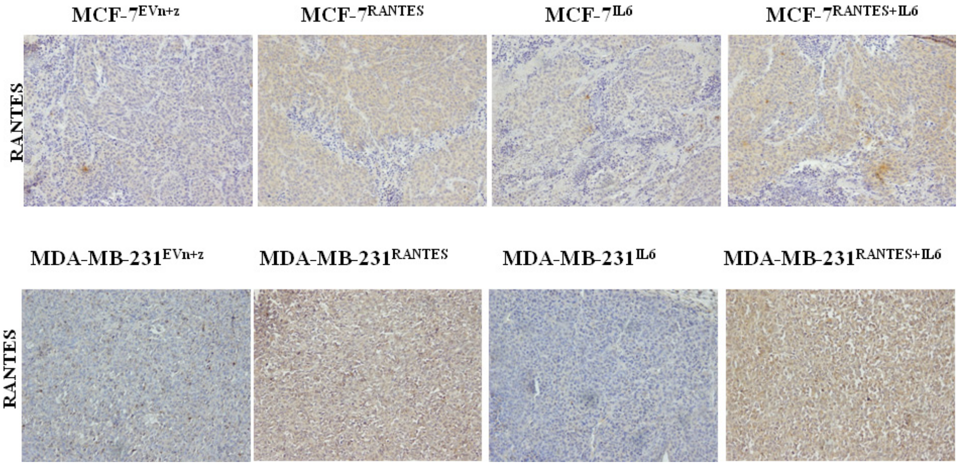

Supplementary Figure 4: immunohistochemical analysis of RANTES expression in mouse xenografts. The expression of RANTES in mammary tumors derived from mice injected with MCF-7 and MDA-MB-231 stable transfectants was evaluated by immunohistochemical analysis. Representative images at 20× are shown. EVn: cells transfected with the empty vector (neomycin); EVz: cells transfected with the empty vector (zeocin); EVn+z: cells transfected with both empty vectors.

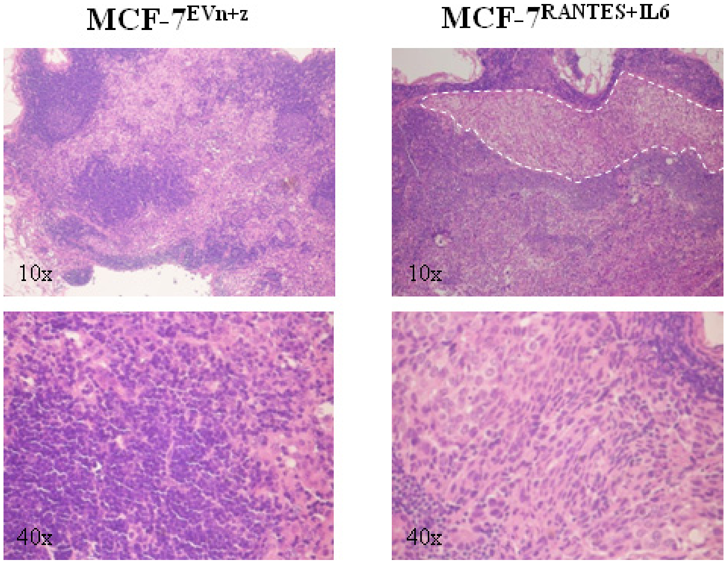

Supplementary Figure 5: Analysis of lymph node metastases in mice injected with MCF7$^{RANTES+IL6}$ and MCF7^{EVn+z} cells. Representative images at 10× (upper panels) and 40× (lower panels) magnifications of inguinal lymph nodes sections stained with hematoxylin and eosin. The white dotted line evidences the metastasis in the lymph node derived from MCF7$^{RANTES+IL6}$ cells. No metastases were observed in the lymph node derived from MCF7 $^{EVn+z}$ cells. EV: empty vector; n: neomycin; z: zeocin.

Supplementary Table 1: Levels of expression of RANTES and IL-6 in the conditioned media from MCF-7 and MDA-MB-231 parental cells

Cell Line	RANTES (pg/48 h/10^5 cells) (mean ± SEM)	IL-6 (pg/48 h/10^5 cells) (mean ± SEM)
MCF-7	14.65 ± 0.01	0.15 ± 0.07
MDA-MB-231	1.67 ± 0.03	1540.83 ± 15.74

values were referred to 1×10^5 cells as determined on the harvesting day, 48 h after seeding. The data presented above were obtained from three independent measurements.
